# Training School-Based Health Clinicians in New Mexico Regarding Adverse Childhood Experiences

**DOI:** 10.3390/healthcare13060638

**Published:** 2025-03-14

**Authors:** Joanna G. Katzman, Laura E. Tomedi, Krishna Chari, Navin Pandey, Anilla Del Fabbro, Mary Ramos, Briana Kazhe-Dominguez

**Affiliations:** 1 Department of Neurosurgery, School of Medicine, Health Sciences Center, University of New Mexico, Albuquerque, NM 87131, USA; 2 College of Population Health, Health Science Center, University of New Mexico, Albuquerque, NM 87131, USA; ltomedi@salud.unm.edu; 3 Project ECHO, Health Sciences Center, University of New Mexico, Albuquerque, NM 87131, USA; tcdr.chari@gmail.com (K.C.); napandey@salud.unm.edu (N.P.); bkazhedominguez@salud.unm.edu (B.K.-D.); 4 Department of Psychiatry, Virginia Tech University, Roanoke, VA 24014, USA; anilladf@gmail.com; 5 School of Medicine, College of Population Health, Health Sciences Center, University of New Mexico, Albuquerque, NM 87131, USA; mramos@salud.unm.edu

**Keywords:** mental health, telementoring, adverse childhood experiences (ACEs), school-based health centers (SBHCs), Project ECHO, community, culturally sensitive

## Abstract

**Background:** Adverse childhood experiences (ACEs) are potentially traumatic experiences that may promote poor mental health, including substance use and suicidality, as well as chronic pain. Telementoring may be used to provide education to school-based health center (SBHC) clinicians and other health professionals in the community to identify and support youth with ACEs. **Methods:** This study was an evaluation of the novel ACEs ECHO telementoring program, which incorporates didactics, case-based learning, and a community of practice to serve school-based health clinicians in New Mexico, a rural state with a high prevalence of ACEs. **Results:** In the program’s first two years, there were 704 unique participants, including SBHC clinicians from 25 of New Mexico’s 33 counties. The pre/post survey demonstrated that the participants reported increases in knowledge in identifying children that experienced ACEs (4.3 versus 3.7, *p* = 0.001) and confidence in supporting children who may be at high risk (4.1 versus 3.3, *p* = 0.001) compared with before they began attending the ACEs ECHO program. The participants also reported that they felt more hopeful that they could help youth with ACEs (4.2 versus 3.3, *p* = 0.001). **Conclusions:** The ACEs ECHO telementoring program may be considered for other rural states and globally as a capacity-building model to educate SBHC clinicians and other health professionals to identify youth at risk for adverse childhood experiences.

## 1. Introduction

New Mexico is the fifth largest and one of the most rural states in the US [1]. Approximately 60 percent of New Mexico’s 2 million population live in rural areas [2]. The poverty rate in New Mexico is 21.2 percent, and 30 of New Mexico’s 33 counties have health professional shortages [2,3,4]. In 2022, New Mexico released a Health Advisory Network (HAN) stating that adverse childhood experiences (ACEs) were the highest in New Mexico [5]. And, according to the last edition of Kids Count, New Mexico ranked 50 out of 50 for child wellbeing [6].

The primary goal of this evaluation was to learn whether school-based health clinicians can increase their knowledge, improve their self-efficacy, and/or improve their skills regarding youth with ACEs after attending the ACEs ECHO telementoring program [7].

## 2. Adverse Childhood Experiences

Adverse childhood experiences (ACEs) are potentially traumatic and stigmatizing experiences of abuse, neglect, violence, and household dysfunction that occur before the age of 18 [8]. These experiences have a lasting impact on mental, physical, and behavioral health. The higher the number of ACEs among respondents, the greater the likelihood and severity of chronic health conditions and premature death. The original ACEs study in 1995 was a household survey from Kaiser Permanente of more than nine thousand respondents and demonstrated a dose–response relationship between childhood trauma, medical illness, and substance use in adult life [8,9,10,11]. This Kaiser study was replicated both nationally and internationally. Especially for low-and-middle-income countries, ACEs remain one of the biggest risk factors for poor mental health [11]. Fortunately, many youth mental health telementoring and telehealth programs showed that rural communities may benefit from virtual connections [12,13,14].

Having multiple adverse childhood experiences increases the risk of poor mental health, including anxiety, depression, substance use, and suicide [11,15,16]. Moreover, the greater number of ACEs in combination with the age of onset of substance use, is predictive of the severity of substance use in some studies [17,18]. Childhood neglect, as well as emotional, physical, and sexual abuse, have all been linked to an increased risk of opioid use disorder [19]. The social determinants of health, including poverty, food, and housing insecurity, as well as limited access to behavioral health care, moderate and contribute to these psychological and behavioral health risks [20].

New Mexico children and adolescents experience one of the highest rates of adverse childhood experiences in the US [21]. In New Mexico, 21.1% of children aged 0 to 17 have experienced at least one ACE, and more than one-quarter (26.5%) have experienced two or more ACEs, the highest such prevalence in the United States (US) [22]. In parallel with the high prevalence of ACEs, drug overdose deaths among youth continue to rise in New Mexico [23], and 10.4% of high school students in New Mexico reported attempting suicide in 2021 [24]. Across ages, the suicide rate in New Mexico has consistently exceeded the national average. In 2021, for example, the age-adjusted suicide rate in New Mexico was 72% higher than the US age-adjusted rate [25].

Clinicians at school-based health centers (SBHCs), and school personnel (school nurses, school counselors, school social workers, teachers, school administrators) are well positioned to assess at-risk youth and provide evidence-based practices and services through integrated, culturally informed interventions. Training school-based health staff and school personnel to identify and respond to early adversity risks and social determinants of health (SDOHs), such as high rates of poverty, food insecurity, lack of optimal health care access, and high rates of ACEs, such as substance use in the home and parental neglect and abuse. Therefore, the ACEs ECHO telementoring program in New Mexico, brings expertise to a large rural state with high rates of child mental health needs, combined with high rates of health professional shortages.

## 3. Project ECHO

The Extension for Community Healthcare Outcomes (ECHO) is a structured tele-mentoring educational model that uses case-based learning to train community providers in specialty care in rural and underserved areas [26]. Project ECHO was shown to improve clinician behaviors and knowledge and reduce treatment disparities in rural and urban underserved communities [27]. ECHO programs use a “hub and spoke” model, where a hub team provides specialty expertise and guides the sessions, and the spokes (participants) attend the virtual training but also provide their own expertise and points of view to create an “all-teach-all learn” environment [28].

## 4. Materials and Methods

### 4.1. ACEs ECHO Session Facilitation, Case-Based Learning, Continuing Medical Education

We conducted a mixed-methods evaluation of the ACEs ECHO program using information collected from the program participants (anyone who had participated at least once). Participation was voluntary and participants could begin or end the program at their own volition. During the ACEs ECHO sessions, hub team members (including a pediatric/adolescent physician specialist, a chronic pain physician, an addiction psychiatrist, a child psychiatrist, and a child psychologist) facilitated didactics and case-based learning. For the case-based learning, hub team members created a case form that participants used during the weekly ACEs ECHO sessions. The case form was de-identified and HIPAA compliant. It therefore included no names or protected health information (PHI). The form included the presenter’s concerns and goals for caring for the youth, potential ACEs and SDOH experienced by the youth, and protective factors in the youth’s life (e.g., self-esteem and appropriate structure, such as routine meals/bedtime). The adverse childhood experiences (ACEs) ECHO program incorporated a mix of didactic lectures and case-based learning while creating a community of practice in order to reach clinicians in rural and underserved communities.

Free Continuing Education Units (CEUs) for healthcare professionals were also provided throughout the program. The ACEs ECHO was promoted to school personnel and school-based health staff, including school-based health center (SBHC) clinicians via several routes: from hospitals and clinics throughout New Mexico, via the New Mexico Department of Health, and by word of mouth. The SBHCs were clinics on or near school grounds that provided integrated medical care and behavioral health services to the students at that school or school district. The ACEs ECHO program was also open to all health professionals and educators in New Mexico and surrounding states. All sessions were held virtually using the Zoom platform (https://zoom.us/).

Sessions ran during the school year from October 2022 to May 2023 and September 2023 to May 2024, as the participants were largely school-based. The ACEs ECHO program was separated into two complementary sessions. The All Hands on Deck (AHOD) ECHO was the session every first Thursday of each month from 12 p.m. to 1 p.m Mountain Time. This AHOD session was developed to be the introductory session for the month and appropriate for all health professionals. The Putting Faces to the ACEs (PFA) ECHO sessions, on the second and third Thursdays of each month from 12 p.m. to 1 p.m. Mountain Time, were developed to be more specific to school-based health clinicians and behavioral health providers. Each PFA session included a didactic and a case presentation.

The ACEs ECHO program was planned and evaluated using a mixed-methods approach. This was achieved by (1) conducting a needs assessment using qualitative key informant interviews; (2) the development of a culturally sensitive curriculum; (3) the evaluation of program implementation data; and (4) conducting a retrospective pre/post survey to assess the changes in knowledge, confidence, and attitude among participants in the program.

### 4.2. Curriculum Development

The Year 1 curriculum, based on responses from the needs assessment, started with the basics of how children build a healthy attachment and feel secure; moved through preventing, identifying, and addressing ACEs; and then focused on substance use and poor mental health. The Year 2 curriculum included resiliency and protective factors, trauma-informed systems of care, and the importance of providing compassionate care (Table 1).

### 4.3. Case-Based Learning

In addition to the evidence-based didactics, a real de-identified youth case was presented by a school-based health clinician from a rural New Mexico county. Examples of Year 1 cases are given below.
14-year-old male who has increasing behavioral issues; starting to use illicit substances and has an unstable living situation.13-year-old female with a history of past sexual assault presenting a pattern of fluctuating grades and recent impulsive behavior, including non-suicidal self-harm.15-year-old female raised by grandmother since a young age due to biological mother’s methamphetamine and heroin use and death of biological father. Now youth is pregnant and grandmother asking her to leave the house.

### 4.4. Program Implementation

Participants who registered online for the ACEs ECHO program were asked their name, gender, location, email, organization, whether they work at an SBHC, and their professional credentials. The participants were also asked, if they work in New Mexico, to identify which of the 33 counties they work in. The registration data were collected as self-administered questionnaires deployed via the Zoom platform.

### 4.5. Retrospective Pre-Post Survey

To assess the impact of the ACEs ECHO program at the end of the curriculum each year, we used a brief survey using REDCap 14.5.42 (Research Electronic Data Capture). The survey was open for two weeks (Year 1: 20 April 2023 to 4 May 2023; Year 2: 12 April 2024 to 26 April 2024) and the participants were sent one email reminder during that time. The participants were asked what they liked the most and least about the ACEs ECHO program and whether they had incorporated skills that they learned from the program into their work. The participants were asked to reflect back on their knowledge, confidence, and attitudes regarding the specific aspects of managing ACEs before they started attending the ACEs ECHO program. They were then asked to assess their current knowledge, confidence, and attitudes. Their responses were recorded on a 5-point Likert scale (strongly disagree = 1, slightly disagree = 2, neither agree nor disagree = 3, slightly agree = 4, strongly agree = 5). The retrospective pre/post survey method was used in similar studies and was used here because the participants had different start and end dates for the program [29].

### 4.6. Statistical Analysis

We used the attendance data and registration information to describe the program participation over time and the participant characteristics, respectively. The survey results were matched to the registration to describe participants who responded to the survey and compared with the overall attendance. We used a chi-squared goodness of fitness test to assess whether the survey respondents were reflective of the overall attendance population based on the location (rural/not rural), profession (clinical, behavioral health, educator, other), and whether they worked at a school or school-based health center (yes/no). The differences between the before and after agreement scores were tested using the Wilcoxon signed rank-sum test (*p* < 0.05). The analysis of the attendance and survey data was conducted in R (version 4.3.3) and R studio [30].

This evaluation protocol was reviewed and approved by the University of New Mexico Health Sciences Center Institutional Review Board (#22-320).

## 5. Results

### 5.1. Evaluation of Program Implementation Data

**Figure 1:**
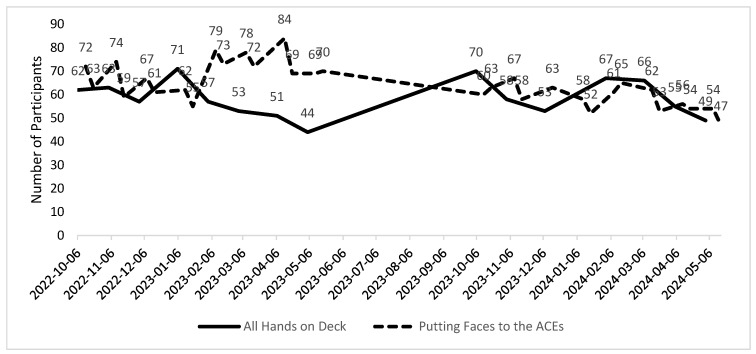
ACEs ECHO program participant attendance, Year 1 and Year 2: 6 October 2022–16 May 2024. Note: no sessions held between 5/6/2023 and 9/6/2023.

Most participants (n = 484 (69%)) who registered online for the ACEs ECHO program were from New Mexico. There was also representation from 28 other states and many non-US countries, including Canada (n = 11) and India (n = 4). Forty-two individuals (6%) who attended at least one ACEs ECHO session in Years 1 and 2 completed a retrospective pre/post survey. There were 704 unique participants in the ACEs ECHO program and 2610 attendances overall for the ACEs ECHO programs, indicating that many participants attended multiple sessions. On average, there were 62 participants per ACEs ECHO session over the last 2 years, which included an average of 58.4 participants per session for the All Hands on Deck sessions and 63.9 participants for the Putting Faces to the ACEs sessions (Figure 1).

The majority of participants were women (73 vs. 12%). Participants were largely healthcare professionals, including behavioral health providers. For example, nurses (9%), clinicians (i.e., doctors or advanced practice clinicians—15%), social workers 15%, mental health professionals 8%, and others (Table 2).

**Figure 2:**
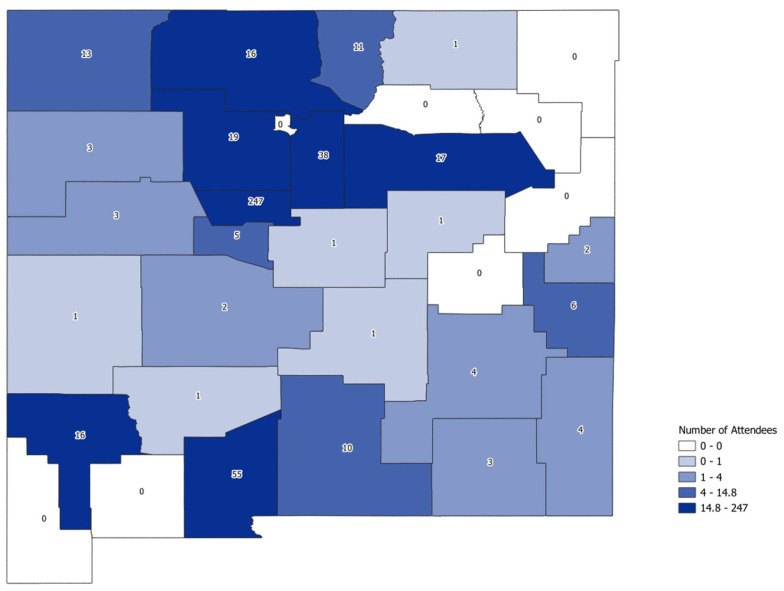
Count of New Mexico attendees by county, Year 1 and Year 2: 6 October 2022 to 16 May 2024.

The participants who answered the question regarding which New Mexico county they worked in—if they resided in New Mexico, were 484 of the 704 unique participants (69%). Although the majority of participants from New Mexico were from Bernalillo, the largest county, 25 of New Mexico’s 33 counties were represented in this program (Figure 2).

### 5.2. Retrospective Pre/Post Survey

A total of 42 people answered the retrospective pre/post survey that was administered at the end of the school years (Year 1 and 2 combined), which reflected a response rate of 67% of participants who attended the last two sessions when the survey was active. The survey respondents were mostly women (n = 40, 95.2%). The respondents were predominantly clinicians (n = 10, 23.8%) and nurses (n = 8, 19.1%), followed by school social workers and school administrators (n = 6, 14.3% each), school counselors (n = 4, 9.5%), and one community health worker (4.8%). Three respondents were missing profession data on their survey. The demographics of attendees who responded to the survey did not differ significantly from the overall attendee demographics (*p* > 0.05).

The participants reported that they perceived significant increases in their knowledge, confidence, and attitudes between when they started attending the sessions and at the end of the school year (Table 3). For example, the largest increase was in agreement with the statement, “I know how to identify a child at risk for developing an opioid use disorder.”. The majority of survey respondents reported that they had incorporated the skills they learned into their work regularly (n = 18, 42.9%) or “a little” (n = 8, 19.1%). Those who incorporated the skills reported several benefits: increased awareness, improved communication, greater attention to detail during inquiries, and a better understanding of parents/guardians dealing with addiction. Additionally, they mentioned directly using the tools and protocols provided, training other school-based health staff, and utilizing the data when discussing patients and their families. When the respondents who reported that they had not incorporated the skills into their work were asked to describe barriers to using the information, they reported it was because they had not encountered the need and that they already were well versed in ACEs (Table 3).

## 6. Discussion

Multiple evidence-based studies showed that ACEs increase the risk of mental health problems, including suicidality, substance use, and chronic pain, along with chronic medical problems, in adulthood [9,10]. The ACEs ECHO team developed a culturally sensitive telementoring program that incorporates evidence-based didactics, case-based learning, and a community of practice among SBHC clinicians and other health professionals throughout most counties of New Mexico to identify ACEs in New Mexico’s youth. The program has had 703 unique participants with 2160 total attendances from October 2022 to May 2024.

This ACEs ECHO program has continued to have excellent participation from rural SBHC clinicians, suggesting that this telementoring program is a valuable part of the day for these school teachers, behavioral health professionals, and other clinicians who are already so busy. In addition, this program, despite operating for over two years, has not seen a reduction in the number of participants attending the weekly virtual sessions. This may further suggest that the ACEs ECHO program is providing value for the attendees. The curriculum, which was developed by the faculty in consultation with key stakeholders and by the participants themselves, continues to remain novel each year, bringing evidence-based information to the participants as well.

Given the rurality and level of poverty in New Mexico, combined with the state’s health professional shortage, the ACEs ECHO program brings a just-in-time solution to many jurisdictions where the part-time school-based health clinician at the town’s local schoolhouse is one of the only health professionals in the county.

Participants in the ACEs ECHO program, for instance, reported that it increased their ability to identify children and adolescents experiencing ACEs, increased their knowledge and confidence in responding to adolescents who are experiencing ACEs, and increased their confidence in referring youth experiencing ACEs to more long-term support services. Additionally, participants reported that they felt more hopeful about their ability to help youth experiencing ACEs.

The novel ACEs ECHO curriculum was developed by the hub team subject matter experts, who incorporated many of the major elements requested by the participants and community stakeholders throughout the state. By addressing topics such as “Trauma- Informed Systems of Care” and “Creating Cultural Compassion”, the participants benefitted from the curriculum, which combined the traditional topics of adverse childhood experiences, substance use, and mental health, in addition to those requested by community practitioners. Developing a thoughtful and deliberate virtual curriculum with cultural sensitivity was critically important for the rural participants of the ACEs ECHO sessions in order to gain their trust and develop a community of practice with mutual respect.

The ACEs ECHO program provides evidence-based techniques and methods for school-based health staff and school personnel to support children at risk of and/or experiencing ACEs. Particularly for health professionals in rural and under-resourced areas, the ACEs ECHO telementoring program has the potential to be capacity building and bring much needed mentorship and support to communities in need [29]. This mentorship has the potential to mitigate burnout, decrease isolation, and provide hope for clinicians and staff so that they can make a meaningful impact with children experiencing ACEs [31]. Afterall, even after two years of programming, there was no decrease in the weekly ACEs ECHO attendance, which averaged 62 participants a session.

There is now a broad body of research documenting the stigmatizing and long-term impact of ACEs on health and wellness. The previous literature also suggests that the early identification of, and intervention with, children experiencing ACEs in the clinical setting could potentially decrease later harm. To date, however, there has been little research on methods to train school-based staff and school personnel on ACEs identification and intervention, especially in rural and underserved communities. This is surprising considering that school-based staff are typically on the front lines of working with children experiencing ACEs [29].

ACEs are not unique to the United States and, in fact, many other countries outside the US may experience even higher rates of adverse childhood experiences [32,33,34]. ACEs may be named “childhood maltreatment” or “childhood adversity” more commonly in some countries. And in low-and-middle-income countries, the manifestations of ACEs may reflect the particular societal surroundings. However, because New Mexico leads the United States in ACEs, and because it is the fifth largest state, scoring very high on the poverty scale, it may resemble some regions outside the US that also experience a high proportion of ACEs [4,5]. Thus, the ACEs ECHO telementoring program may be beneficial, not only in New Mexico but also for other rural and underserved populations in the US, as well as in other countries.

### Strengths and Limitations

Several strengths and limitations in our study warrant consideration. First, our findings may be limited by the specific context and characteristics of the participating schools and health professionals and may not be generalizable to other settings.

Second, many surrounding and nearby states (Colorado, Arizona, Oklahoma, Arkansas, and Oregon) have inquired with our hub team regarding the success of our program, asking for assistance initiating telementoring programs similar to ours for their state, given their similar rurality, ACE, and SDOH levels. Other states, such as California, have performed different types of pilot ACEs projects such as ours, and have also been very successful [31].

Third, although we are not yet able to capture the long-term impact of the ACEs ECHO Project on adolescent health outcomes, our results show promise that telementoring programs can provide support to school-based health staff and increase their capacity to implement evidence-based interventions. Once the state of New Mexico has collected new data on overdoses, mental health, and chronic pain, additional research will be needed to assess the association between the success of the ACEs ECHO program with county-level data among the states’ youth.

Lastly, we used a convenience sample for the survey (all participants who attended at least once were invited to participate). While we compared key characteristics [location (rural/not rural), profession (clinical, behavioral health, educator, other), and whether they worked at a school or school-based health center (yes/no)] of the people who responded to the survey to the characteristics of the overall participant population, people who were more motivated to actively learn may have also been more likely to participate in the survey, which could have biased our results.

In future ACEs ECHO studies, the authors will continue to deploy rigorous implementation research focused on incorporating ACEs intervention training and telementoring into school-based settings, including using Project ECHO. When resources allow, incorporating long-term outcomes and additional behavior change measures could provide additional information on the impact of ongoing telementoring and a community of practice models on addressing ACEs in schools.

## 7. Conclusions

The findings of this study underscore the potential of the Project ECHO telementoring model to support SBHC clinicians and other health professionals in addressing ACEs in rural and underserved settings throughout New Mexico. The capacity-building aspect of the ACEs ECHO telementoring program may be deployed to other rural states with similar healthcare shortages, where there is a growing need to address children at risk.

## Figures and Tables

**Table 1:** Adverse childhood experiences (ACEs) ECHO program curriculum Years 1 and 2 dates and session topics.

Year 1 Curriculum: 6 October 2022 to 18 May 2023	Year 2 Curriculum: 5 October 2023 to 16 May 2024
10/6—Foundations of a Secure Base and Healthy Attachment ^1^	10/5—ACEs: Resiliency and Protective Factors
10/13—How Can a Secure Attachment Protect Children? ^2^	10/12—Legal Rights of Children in New Mexico
10/20—Building the Base: Universal Skills to Build Healthy Relationships with Kids ^2^	10/19—Understanding New Mexico CYFD Processes and Guidelines
11/3—Determinants of Children’s Health: Social, Moral, and Environmental ^1^	11/2—Trauma Informed Systems of Care
11/10—Social Determinants of Health: Influences of Race, Culture, and History ^2^	11/9—Trauma Informed Care in Schools: Integrating Socio-Emotional Learning
11/17—Community Schools: A Capstone Solution to Prevent Adverse Childhood Experiences ^2^	11/16—From Historical Trauma to Trauma Informed Care: An Interview with an Expert
12/1—Adverse Childhood Experiences, Social Determinants of Health, and their Relationship to Complex Trauma ^1^	12/7—Healing Centered Engagement: Moving From “What Happened to You” to “What’s Right with You?”
12/8—Neurobiology and Behaviors After ACEs and Psychological Trauma ^2^	12/14—Healing Addiction in the Community: Serenity Mesa
12/15—How to Identify At-Risk Children and Screen for ACEs: Individuals and Institutional Best Practices ^2^	12/21—No ECHO session
1/5—Suicide and Non-Suicidal Self-Injury (NSSI) in Youth ^1^	1/4—No ECHO session
1/12—Risk Factors and Identification of Children at Risk for Suicide and Non-Suicidal Self-Injury ^2^	1/11—Cultivating Cultural Compassion in New Mexico
1/19—Skills: Assessment, Intervention, and Referrals for Suicide and Non-Suicidal Self-Injury ^2^	1/18—Culturally Sensitive ACEs Interventions with Immigrant Populations
2/2—Substance Use Disorder and Youth: Cannabis, Vaping, and Alcohol ^1^	2/1—Opioid Use Disorder in the Fentanyl Era
2/9—How ACEs Can Increase Risk for and Identification of Children at Risk for Cannabis, Vaping, and Alcohol ^2^	2/8—SBIRT (Screening, Brief Intervention, and Referral to Treatment): A Public Health Approach for Youth
2/16—Skills: Assessment, Intervention, and Referrals for Cannabis, Vaping, and Alcohol ^2^	2/15—Demonstration of Youth Substance Use Interventions
3/2—Substance Use Disorder and Youth: Opioids and Stimulants ^1^	3/7—Supporting Youth Behavioral Health: Post Crisis Event and School Re-entry for Students
3/9—How ACEs Can Increase Risk for and Identification of Children at Risk for Opioids and Stimulants ^2^	3/14—New Mexico Youth Behavioral Health Crises: Warning Signs, Interventions, and Referrals
3/16—Skills: Assessment, Intervention, and Referrals for Opioids and Stimulants ^2^	3/21—SBHC’s Impact on Youth Mental Health
4/6—Explaining the Adverse in ACEs: How Adverse Childhood Experiences (ACEs) Increases the Risk of Chronic Pain, Substance Use Disorder, Mental Health and Suicidality ^1^	4/4—Integrative Pain Management in Youth Panel Discussion
4/13—The Unholy Trinity: How Adverse Childhood Experiences and Social Determinants of Health Can Increase the Risk of Chronic Pain and Substance Use Disorder ^2^	4/11—Case Presentation: Interventions for Chronic Pain with Youth
4/20—Skills: Assessment, Intervention, and Referrals for Chronic Pain in Youth ^2^	4/18—It Takes a Village: Community Interventions for Chronic Pain
5/4—Youth Mental Health: Depression and Anxiety ^1^	5/2—Community Health Workers in School Based Health Centers
5/11—Youth Mental Health and Minority Statuses: Race, Culture, and Sexual Orientation ^2^	5/9—Integrated Care Model
5/18—Skills: Assessment, Intervention, and Referrals for Youth Mental Health ^2^	5/16—The Clinical Reasoning and Case Formulation Model (CRCF)

^1^ All Hands on Deck ECHO. ^2^ Putting Faces to the ACEs ECHO.

**Table 2:** Participant characteristics by ECHO ^1^, adverse childhood experiences (ACEs) ECHO program, 6 October 2022 to 16 May 2024.

Gender	All Hands on Deck, N = 392 n (%)		Putting Faces to the ACEs, N = 579 n (%)		Total N = 704 n (%)
	Y1	Y2	Y1	Y2	
Female	119 (58.6)	160 (84.7)	206 (60.6)	201 (84.1)	514 (73.0)
Male	14 (6.9)	25 (13.2)	35 (10.3)	35 (14.6)	84 (11.9)
Non-binary	1 (0.5)	2 (1.1)	3 (0.9)	1 (0.4)	6 (0.9)
Prefer not to answer	6 (3.0)	2 (1.1)	6 (1.8)	2 (0.8)	10 (1.4)
Missing	63 (31)	0 (0.0)	90 (26.5)	0 (0.0)	90 (12.8)
**Profession**					
Nurse	25 (12.3)	8 (2.9)	55 (15.7)	6 (2.5)	66 (9.4)
Clinician ^2^	25 (12.3)	38 (13.6)	51 (14.5)	33 (13.8)	106 (15.1)
Administrative	23 (11.3)	37 (13.2)	40 (11.4)	30 (12.6)	91 (12.9)
Social worker	20 (9.9)	47 (16.8)	58 (16.5)	42 (17.6)	107 (15.2)
School counselor	10 (4.9)	7 (2.5)	21 (6.0)	4 (1.7)	38 (5.4)
Public health professional	20 (9.9)	12 (4.3)	23 (6.6)	10 (4.2)	39 (5.5)
Mental health professional	11 (5.4)	35 (12.5)	12 (3.4)	32 (13.4)	58 (8.2)
Teacher	12 (5.9)	12 (4.3)	12 (3.4)	8 (3.3)	29 (4.1)
Allied health professional ^3^	5 (2.5)	18 (6.4)	6 (1.7)	17 (7.1)	25 (3.6)
Community health worker	3 (1.5)	17 (6.1)	4 (1.1)	17 (7.1)	23 (3.3)
Other	23 (11.3)	47 (16.8)	48 (13.7)	38 (15.9)	88 (12.5)
Missing	26 (12.8)	2 (0.7)	21 (6.0)	2 (0.8)	34 (4.8)

^1^ ECHO programs were not mutually exclusive and may not add up to the total. ^2^ Doctor, nurse practitioner, and physician assistant. ^3^ Occupational therapists, physical therapists, radiographers, respiratory therapists, and speech language pathologists.

**Table 3:** Changes in participants’ knowledge, confidence, and attitude, adverse childhood experiences (ACEs) ECHO program ^1^, 6 October 2022 to 16 May 2024, N = 42.

Learning Objective	Before Score, Mean (SE)	After Score, Mean (SE)	*p*-Value ^2^
I know how to identify children with Adverse Childhood Experiences (ACEs)	3.7 (0.2)	4.3 (0.1)	<0.0001
I know how to identify protective factors for children experiencing ACEs.	3.6 (0.2)	4.3 (0.1)	<0.0001
I know how to begin a difficult conversation with a child experiencing ACEs.	3.3 (0.2)	4.2 (0.1)	<0.0001
I know how to begin a difficult conversation with the parents or guardians of a child experiencing ACEs.	3.2 (0.2)	4.1 (0.2)	<0.0001
I know how to identify a child at risk for developing an opioid use disorder.	2.9 (0.2)	4 (0.2)	<0.0001
I feel confident that I could respond to, and treat a child using illicit substances and alcohol.	3.2 (0.2)	4 (0.2)	<0.0001
I feel confident that I could respond to, and treat a child experiencing mental health problems (e.g., suicidality and non-suicidal self-harm).	3.3 (0.2)	4.1 (0.2)	<0.0001
I am confident that I know where to refer children experiencing ACEs who needed behavioral health services.	3.4 (0.2)	4.3 (0.1)	<0.0001
I am very knowledgeable on the subject of normal attachment in children.	3.1 (0.2)	4.2 (0.2)	<0.0001
I am very knowledgeable on how social determinants of health can interact with ACEs.	3.4 (0.2)	4.1 (0.2)	<0.0001
I am confident in my ability to support children who may be at higher risk for ACEs (e.g., gender expansive children).	3.3 (0.2)	4.1 (0.3)	<0.0001
I feel hopeful that I could help children experiencing ACEs.	3.3 (0.3)	4.2 (0.2)	<0.0001

^1^ Agreement scores: strongly disagree = 1, slightly disagree = 2, neither agree nor disagree = 3, slightly agree = 4, strongly agree = 5. ^2^ Differences between before and after tested using Wilcoxon signed rank-sum test.

## Data Availability

The data and materials from this study are not publicly available.
